# Rapid Determination of Cr^3+^ and Mn^2+^ in Water Using Laser-Induced Breakdown Spectroscopy Combined with Filter Paper Modified with Gold Nanoclusters

**DOI:** 10.3390/bios14060267

**Published:** 2024-05-23

**Authors:** Xuan Dai, Changbo Song, Shixiang Ma, Fengjing Cao, Daming Dong

**Affiliations:** 1 School of Mechanical Engineering, Guangxi University, Nanning 530004, China; daix@st.gxu.edu.cn (X.D.); dongdm@nercita.org.cn (D.D.); 2 Research Center of Intelligent Equipment, Beijing Academy of Agriculture and Forestry Sciences, Beijing 100097, China; s1270772622@163.com (C.S.); masx@nercita.org.cn (S.M.)

**Keywords:** laser-induced breakdown spectroscopy, Au nanoclusters, heavy metal determination, paper test, chromium, manganese

## Abstract

Excessive emissions of heavy metals not only cause environmental pollution but also pose a direct threat to human health. Therefore, rapid and accurate detection of heavy metals in the environment is of great significance. Herein, we propose a method based on laser-induced breakdown spectroscopy (LIBS) combined with filter paper modified with bovine serum albumin-protected gold nanoclusters (LIBS-FP-AuNCs) for the rapid and sensitive detection of Cr^3+^ and Mn^2+^. The filter paper modified with AuNCs was used to selectively enrich Cr^3+^ and Mn^2+^. Combined with the multi-element detection capability of LIBS, this method achieved the simultaneous rapid detection of Cr^3+^ and Mn^2+^. Both elements showed linear ranges for concentrations of 10–1000 μg L^−1^, with limits of detection of 7.5 and 9.0 μg L^−1^ for Cr^3+^ and Mn^2+^, respectively. This method was successfully applied to the determination of Cr^3+^ and Mn^2+^ in real water samples, with satisfactory recoveries ranging from 94.6% to 105.1%. This method has potential application in the analysis of heavy metal pollution.

## 1. Introduction

In recent years, heavy metal pollution has become increasingly severe owing to industrial production, mineral extraction, human activities, and the lack of efficient control and treatment methods [1,2,3]. Notably, heavy metals (Cr, Mn, Cd, Hg, Pb) are nondegradable, leading to their ongoing accumulation in the environment, which presents a long-term threat [4,5]. These heavy metals can enter the human body through a wide variety of pathways (e.g., dermal contact, ingestion, inhalation), posing risks to human health [6,7]. For example, manganese (Mn) is an essential trace element for the human body, but excessive intake can result in neurological disorders [8,9]. Chromium (Cr) may cause hemolysis, renal failure, liver failure, and lead to various types of malignant tumors, particularly lung cancer [10]. Hence, to reduce the environmental and human health risks related to heavy metal pollution, it is crucial to have a method for the rapid and sensitive detection of Cr^3+^ and Mn^2+^.

The common, extensively used methods for heavy metal detection are atomic absorption spectrometry (AAS) [11,12], atomic fluorescence spectrometry (AFS) [13,14], inductively coupled plasma mass spectrometry (ICP-MS) [15,16], and inductively coupled plasma optical emission spectroscopy (ICP-OES) [17,18]. Although these techniques are capable of sensitive detection, they are unsuitable for rapid on-site detection due to requiring sample pretreatment, time-consuming procedures, labor-intensive requirements, and high costs. Laser-induced breakdown spectroscopy (LIBS) is a novel atomic emission spectroscopy that uses a high-energy pulse laser to ablate and break down the sample surface to generate plasma, and then analyzes the plasma emission spectrum to determine the elemental composition and content [19,20]. LIBS offers several unique benefits, including multi-element analysis, fast response (microseconds), minimal sample treatment, and non-contact measurement [21]. Thus, it has become a powerful analytical technique for heavy metal detection. Yi et al. used LIBS assisted by a solid–liquid–solid transformation method to detect cadmium (Cd) and lead (Pb) in soil, with limits of detection (LOD) of 0.067 and 0.94 mg kg^−1^, respectively [22]. Zhu et al. used LIBS assisted by laser-induced fluorescence to detect Pb with an LOD of 0.054 mg kg^−1^ in natural rhododendron leaf samples [23]. Moreover, LIBS has multiform (solid, liquid, gas) detection capability. Nevertheless, liquid analysis by LIBS is associated with several inherent drawbacks, such as splashing, surface ripples, extinction of emitted intensity, and a shorter plasma lifetime, which would greatly reduce the laser ablation efficiency, signal repeatability, and quantitative accuracy [24]. To solve these problems, many methods have been proposed, including water jets, freezing samples, and employing a double pulse with different geometric positions [25]. However, these techniques require extra equipment, which increases the complexity and analytical cost. Matrix transformation is a frequently used strategy for conquering the limitations of LIBS for the analysis of liquid samples. Díaz Pace et al. converted liquid solutions into solid pellets of calcium hydroxide by mixing with calcium oxide to successfully detect Cr, Pb, Cd, and zinc (Zn) in liquid samples [26]. Therefore, converting liquid samples into a solid matrix is a feasible method to overcome the shortcomings of liquid analysis and improve the detection sensitivity and stability.

There have been many studies of heavy metal detection using nanoparticles. These studies primarily focused on exploiting the changes in the physical and chemical properties (e.g., color, fluorescence, surface plasmon resonance) of nanoparticles induced by heavy metals to achieve the purpose of detection [27,28,29]. However, these signal readout methods are susceptible to interference from other substances, and non-specific aggregation of nanoparticles may occur [30]. Therefore, it is worth noting that many nanoparticles have the ability to adsorb heavy metals. This capability can be utilized as a means of enriching heavy metals in water for detection, thereby avoiding these problems [31]. Protein-protected metal nanoclusters (PPMNCs) are a novel nanomaterial [32]. Related studies have utilized PPMNCs for the adsorption of heavy metals. Bi et al. synthesized MNP/ATT-AuNCs@ZIF-8, which was used for the simultaneous detection and removal of mercury (Hg), with a short capture time (20 min), high removal efficiency (>96.9%), and excellent reusability (10 cycles) [33]. Compared with common nanoparticles, PPMNCs have a smaller size (<10 nm) and larger specific surface area. These characteristics provide numerous active sites for the adsorption of heavy metals. Moreover, their protein-templated synthesis method not only ensures precise control of the size and shape but is also environmentally friendly. In addition, the protective shell of the protein makes this material highly biocompatible. Therefore, PPMNCs show great potential in the detection and removal of heavy metals from the environment.

In this study, we developed a rapid and sensitive method based on LIBS combined with filter paper modified with bovine serum albumin-protected Au nanoclusters (LIBS-FP-AuNCs) for the simultaneous detection of Cr^3+^ and Mn^2+^. The filter paper was modified with AuNCs by soaking in this solution for 1 h. Owing to the adsorption capabilities of AuNCs, the modified filter paper showed the rapid enrichment and separation of Cr^3+^ and Mn^2+^ from the sample by liquid–solid conversion in 10 min. Direct determination was completed using LIBS. Therefore, the simultaneous rapid detection of Cr^3+^ and Mn^2+^ could be performed using this method, and it is cost-effective and simple to operate. Furthermore, when combined with portable LIBS instruments, it holds great promise for the on-site detection of heavy metals.

## 2. Materials and Methods

### 2.1. Materials and Instrumentation

Chloroauric acid (HAuCl_4_·3H_2_O) was purchased from Beijing Chemical Works (Beijing, China). Sodium hydroxide (NaOH) was purchased from Bolinda Technology (Shenzhen, China). Bovine serum albumin (BSA) and trisodium citrate were purchased from Sigma-Aldrich (Shanghai, China). The filter papers were purchased from Aoke (Taizhou, China). The standard solutions (K^+^, Cr^3+^, Cr^6+^, Mn^2+^, Ni^2+^, Cu^2+^, Zn^2+^, Cd^2+^, Hg^2+^, and Pb^2+^) were obtained from the China Standard Material Network. The Hg^2+^ standard solution was 100 mg L^−1^, and the other ion standard solutions were 1000 mg L^−1^. All the chemicals were of analytical purity and were used without further purification.

We employed scanning electron microscopy (SEM; JSM-7800 F, JEOL, Tokyo, Japan) and transmission electron microscopy (TEM, SU-8220, Hitachi, Tokyo, Japan) for the characterization of AuNCs and AuNPs. We employed ICP-MS (Prodigy 7, Teledyne Leeman Labs, Hudson, NH, USA) to detect the concentrations of Cr^3+^ and Mn^2+^ in real samples.

**Figure 1:**
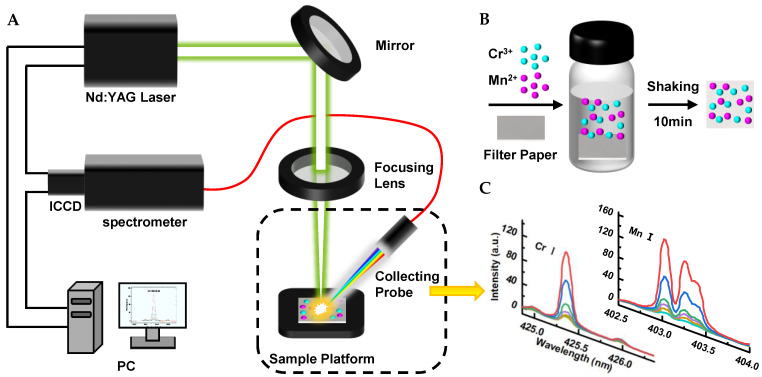
Schematic illustration of the LIBS-FP-AuNCs sensor for heavy metal detection: (**A**) schematic diagram of the LIBS system; (**B**) schematic diagram of the heavy metal adsorption by AuNCs-modified filter paper; and (**C**) spectra of Cr and Mn.

The LIBS system that we used is shown in Figure 1A. A Q-Switch Nd: YAG laser (wavelength 532 nm, repetition rate 20 Hz, pulse width 7 ns) was used to generate a high-energy pulsed laser beam. The laser beam was reflected by a mirror and then focused on the filter paper surface through a focusing lens (*f* = 25 mm). The laser beam ablated the sample surface to generate a plasma signal. The plasma signal was collected through a collecting probe and coupled to a spectrometer (Shamrock 500i, grating 2400 L mm^−1^, slit width 200 μm; Andor Tech, Belfast, UK) equipped with an intensified charge-coupled device (ICCD, iStar 320T: DH320T-18F-E3-26 mm; Andor Tech) through an optical fiber. The LIBS system had a detection range of about 8 nm at around 400 nm. The collected data were analyzed using a computer. A three-dimensional sample platform was used to adjust the position of the laser ablation for the optimal signal.

### 2.2. Preparation of AuNCs and AuNPs Modified Filter Paper

According to a previous study [32], AuNCs were prepared using BSA as a template for the reduction and stabilization of gold atoms in subnanometer-sized clusters. Briefly, we added 2.5 mL of chloroauric acid solution (10 mM, 37 °C) to 2.5 mL of BSA solution (50 mg mL^−1^, 37 °C) under vigorous shaking. After 2 min, 500 μL of NaOH solution (1 M) was added and the mixture was shaken at 37 °C for 12 h. During this period, a color change from light yellow to deep yellow, and then to dark brown, was observed in the reaction mixture.

**Figure 2:**
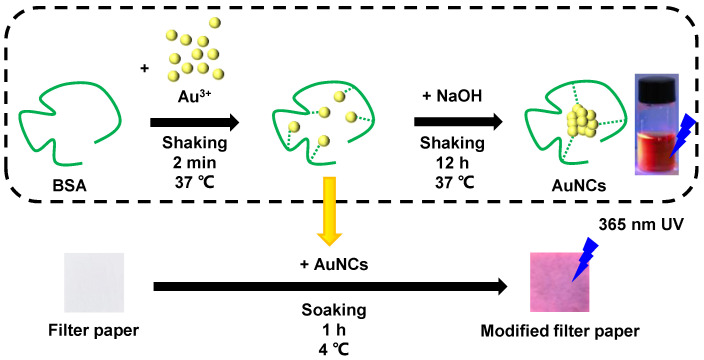
Schematic diagram of the preparation of the AuNCs-modified filter papers, including the synthesis of AuNCs and the modification of filter papers. The AuNCs solution and the AuNCs-modified filter paper emitted red fluorescence under 365 nm ultraviolet light.

Filter papers modified with AuNCs were subsequently prepared. Twenty pieces of filter paper (10 mm × 10 mm) were placed in the AuNCs solution and soaked for 1 h. The filter papers were then removed, rinsed three times with deionized water, and air-dried at room temperature for 1 h. The solution and filter paper showed an intense red fluorescence under 365 nm ultraviolet light (UV), confirming the successful reaction and modification of AuNCs. The AuNCs-modified filter papers were stored at 4 °C in an airtight container for further use. A schematic diagram of the preparation of the AuNCs-modified filter papers is shown in Figure 2. The preparation of the AuNPs-modified filter papers used trisodium citrate as the reductant, as previously described [30].

### 2.3. LIBS-FP-AuNCs Sensor for Cr^3+^ and Mn^2+^ Detection

For Cr^3+^ and Mn^2+^ detection, we established a procedure using the LIBS-FP-AuNCs sensor. As shown in Figure 1B, to adsorb the target ions using the FP-AuNCs, we first prepared a 4 mL Cr^3+^ and Mn^2+^ solution, placed a piece of FP-AuNCs in the solution, and then agitated it for 10 min on a shaker. The filter paper was removed from the solution, rinsed three times with deionized water, and dried using a heating platform at 50 °C for 2 min. The filter paper was then attached to a glass slide using double-sided tape and positioned on the sample platform for LIBS measurement.

To improve the sensitivity of Cr^3+^ and Mn^2+^ detection, we optimized the experimental parameters. The energy of the Nd: YAG laser was 50 mJ, the spot size of the focused laser was ~150 μm, and the delay time and gate width of the spectrometer were both 4 μs. The selected analytical lines were Cr I 425.43 nm and Mn I 403.08 nm. The spectra of Cr and Mn are shown in Figure 1C.

## 3. Results and Discussion

### 3.1. Comparison of Adsorption Capabilities of Filter Papers Modified with Different Materials

**Figure 3:**
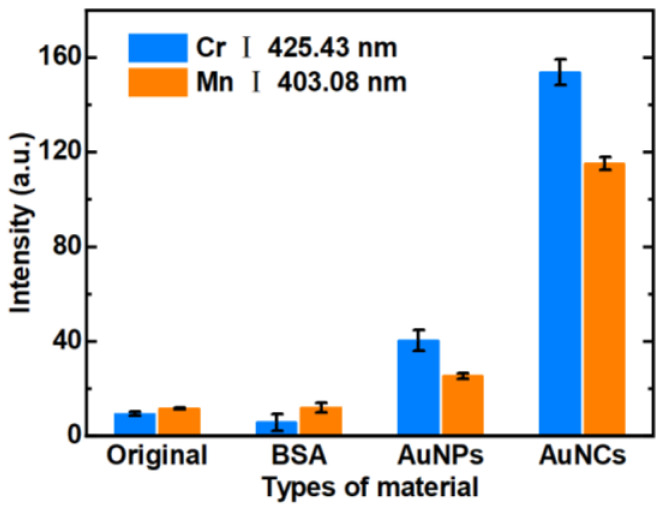
Spectral intensities of Cr and Mn using filter papers modified with different materials.

To investigate the optimal adsorption capability and mechanism of AuNCs for Cr^3+^ and Mn^2+^, we conducted a comparative experiment to evaluate the spectral response characteristics of filter papers modified with different materials for the detection of the target ions. An original filter paper, BSA-modified filter paper, and AuNPs-modified filter paper were prepared and tested, and they were then compared with the AuNCs-modified filter paper. The experimental results are depicted in Figure 3. It can be clearly seen that the spectral intensities of Cr^3+^ and Mn^2+^ were weak when using the original filter paper and that modified with BSA. This indicated that these had no obvious adsorption capacities for Cr^3+^ and Mn^2+^. Conversely, enhanced spectral signals of Cr^3+^ and Mn^2+^ were observed when the AuNPs-modified filter paper was used, demonstrating that AuNPs have a certain degree of adsorption capacity for these ions but were unable to achieve the purpose of efficient adsorption. The use of the AuNCs-modified filter paper showed the perfect spectral intensities for Cr^3+^ and Mn^2+^. Compared with the AuNPs-modified filter paper, there were significant increases in the spectral intensities of Cr^3+^ and Mn^2+^ by 3.8-fold and 4.5-fold, respectively. The possible reasons for these significant enhancements were hypothesized as (1) the smaller size of AuNCs, resulting in a larger specific surface area, which provides more active sites for the adsorption of heavy metals; and (2) the carboxyl groups (–COOH) of BSA on the surface of AuNCs, which provide a very strong affinity between Cr^3+^ and –COOH. [34,35]. In addition, Mn follows Cr in the Periodic Table, so it may induce a similar interaction between Mn^2+^ and AuNCs.

**Figure 4:**
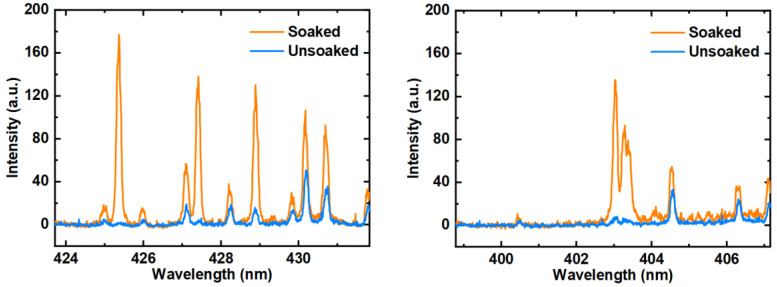
LIBS spectra of AuNCs-modified filter paper soaked and unsoaked in Cr^3+^ and Mn^2+^ solution.

We compare the spectra of the AuNCs-modified filter paper soaked and unsoaked in Cr^3+^ and Mn^2+^ solution in Figure 4. The elements detected on the unsoaked filter paper were Fe (Fe I 404.58 nm, Fe I 425.08 nm, Fe I 406.36 nm, Fe I 427.18 nm, Fe I 428.24 nm) and Ca (Ca I 430.25 nm, Ca I 430.77 nm). It can be seen that the spectral peaks of the Cr and Mn elements were not detected on the unsoaked filter paper. After being soaked, the elements detected were also Cr (Cr I 425.43 nm, Cr I 427.48 nm, Cr I 428.97 nm) and Mn (Mn I 403.08 nm, Mn I 403.31 nm, Mn I 403.45 nm). The comparison showed that Cr^3+^ and Mn^2+^ were absorbed.

### 3.2. Characterization of AuNCs-Modified Filter Paper

**Figure 5:**
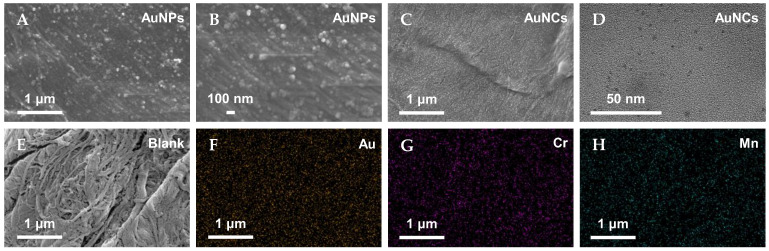
(**A**,**B**) SEM images of filter paper modified with AuNPs, (**C**,**D**) SEM and TEM images of AuNCs, and (**E**) SEM image of unmodified filter paper. (**F**–**H**) Elemental mapping images of FP-AuNCs after adsorption of Cr^3+^ and Mn^2+^.

To verify the better adsorption capacity of FP-AuNCs for Cr^3+^ and Mn^2+^, we used SEM to characterize the original filter paper and those modified with the different nanomaterials, and we used TEM to characterize the AuNCs. As shown in Figure 5A–E, we could observe more nanoparticles fixed on the filter paper surface modified with AuNCs than on that with AuNPs. The particle sizes of the AuNCs on the filter paper were less than 10 nm, which is smaller than those of the AuNPs (approximately 30–50 nm). Therefore, the filter paper modified with AuNCs had better heavy metal adsorption capability due to the larger specific surface area of AuNCs and greater number of AuNCs fixed on the filter paper, resulting in more active sites available for target ion adsorption.

An energy dispersive spectrometer (EDS) was employed to characterize the element mapping of the AuNCs-modified filter paper after adsorption of Cr^3+^ and Mn^2+^. The results are shown in Figure 5F–H. Au was uniformly distributed on the surface of the filter paper, which confirmed the successful modification of the filter paper by the AuNCs. Moreover, the detection of Cr and Mn on the surface of the filter paper suggests that AuNCs exhibit excellent capability for adsorbing these two elements.

### 3.3. Optimization of Experimental Conditions

**Figure 6:**
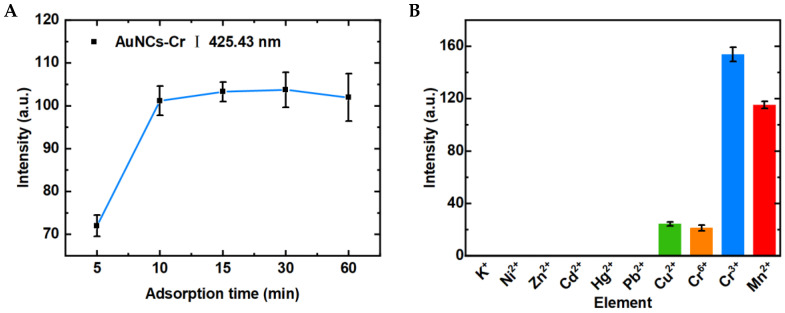
(**A**) Optimization of the adsorption time of Cr^3+^ on the FP-AuNCs. (**B**) Spectral intensities of different elements using LIBS-FP-AuNCs.

We optimized the adsorption time of the FP-AuNCs to ensure the rapid detection of Cr^3+^ and Mn^2+^ in the solution. Taking Cr^3+^ as an example, the adsorption times in Cr^3+^ solution (4 mL, 200 μg L^−1^) were set to 5, 10, 15, 30, and 60 min. The FP-AuNCs were measured by LIBS after adsorption and the spectral intensities were obtained. The results are shown in Figure 6A. The spectral intensity of Cr^3+^ increased from 5 to 10 min, and it reached a peak at 10 min. The spectral intensities remained stable after 10 min, indicating that the best adsorption process for Cr^3+^ was completed within 10 min. Therefore, we set 10 min as the final adsorption time in subsequent experiments.

### 3.4. LIBS-FP-AuNCs for Multi-Element Detection

The selectivity of LIBS-FP-AuNCs was subsequently evaluated. We selected and detected 10 common metal ions (K^+^, Cr^3+^, Cr^6+^, Mn^2+^, Ni^2+^, Cu^2+^, Zn^2+^, Cd^2+^, Hg^2+^, and Pb^2+^, each at 200 μg L^−1^) in environmental water under the same experimental conditions. The optimal spectral lines of the 10 ions were selected (K I 766.49 nm, Cr I 425.43 nm, Mn I 403.08 nm, Ni I 403.08 nm, Cu I 425.43 nm, Zn I 403.08 nm, Cd I 403.08 nm, Hg I 435.83 nm, Pb I 405.78 nm), and their spectral intensities are shown in Figure 6B. The LIBS-FP-AuNCs demonstrated obvious spectral signals for Cr^3+^ and Mn^2+^, whereas the spectral signals for Cr^6+^ and Cu^2+^ were too weak to meet the requirements for trace detection. Other ions, including K^+^, Ni^2+^, and Zn^2+^, were not detected using the FP-AuNCs. These results indicate that the AuNCs-modified filter paper has highly selective adsorption for Cr^3+^ and Mn^2+^.

### 3.5. Detection Sensitivity of LIBS-FP-AuNCs for Cr^3+^ and Mn^2+^ Analysis

**Figure 7:**
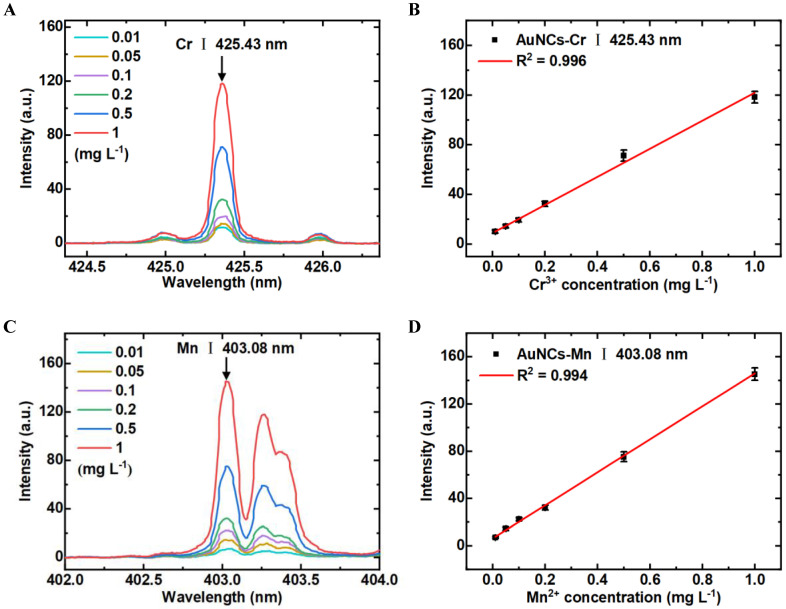
LIBS spectra under different concentrations of Cr^3+^ (**A**) and Mn^2+^ (**B**). Relationship of Cr^3+^ (**C**) and Mn^2+^ (**D**) between the spectral intensities and the concentrations detected using LIBS-FP-AuNCs.

To assess the sensitivities of LIBS-FP-AuNCs in detecting Cr^3+^ and Mn^2+^, we examined the spectra of Cr^3+^ and Mn^2+^ at different concentrations. Each sample was measured ten times by surface scanning by one pulse per point and 2 mm distance between the successive points, and then the average intensity calculated. Quantification was performed using the coefficient of determination (*R*^2^) and LOD. Figure 7A,C show the spectra of Cr^3+^ and Mn^2+^ at different concentrations obtained by LIBS-FP-AuNCs. The spectral intensity at the characteristic peak of 425.43 nm increased with the concentration of Cr^3+^ from 0.01 to 1 mg L^−1^; the same trend was observed for Mn^2+^. We established a positive correlation between the intensity of the characteristic spectral line and the ion concentration. To eliminate the impact of random error, we constructed the calibration curve between the ΔLIBS intensity (the intensity of sample minus the background noise) and the heavy metal ion concentrations. Figure 7B,D show the calibration curves for Cr^3+^ and Mn^2+^ in a series of gradient aqueous solutions obtained by LIBS-FP-AuNCs. The linear ranges were 0.01–1 mg L^−1^ for both Cr^3+^ and Mn^2+^. The *R*^2^ were 0.996 and 0.994 for Cr^3+^ and Mn^2+^, respectively. The LOD was calculated to measure the sensitivity using the formula LOD = 3 *S*/*k*, where *S* is the standard deviation of the spectral background noise and *k* is the slope of the calibration curve. Through calculation, the LODs were 7.4 μg L^−1^ for Cr^3+^ and 9.0 μg L^−1^ for Mn^2+^, which are lower than the respective limits in Chinese standards for drinking water quality (50 μg L^−1^ Cr, 100 μg L^−1^ Mn).

**Figure 8:**
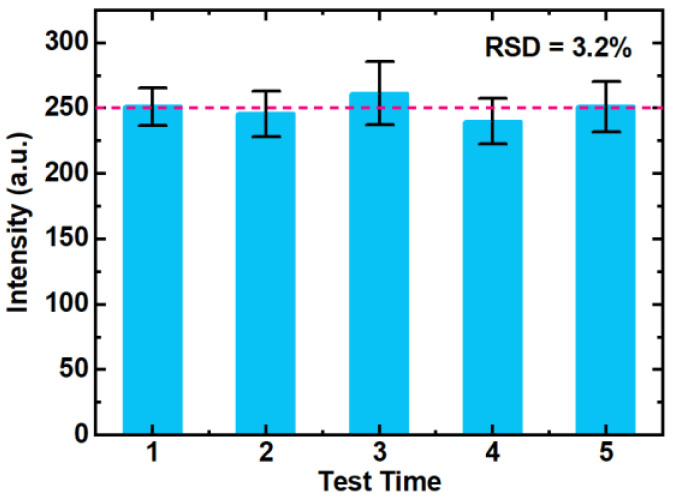
Cr spectral intensities of five filter papers modified with AuNCs.

To illustrate the signal stability and reproducibility of the LIBS-FP-AuNCs method, we detected five pieces of AuNCs-modified filter paper that had adsorbed Cr^3+^ at the same concentration (200 μg L^−1^). Five different positions on the filter paper were randomly selected to record the LIBS signals, and the average spectrum was obtained. The average spectral intensities of the five filter papers are shown in Figure 8. The relative standard deviation (RSD; 3.2%) was calculated based on the average spectral intensity of the five filter papers. This demonstrates that the LIBS-FP-AuNCs method possesses favorable reproducibility for Cr^3+^ and Mn^2+^ analysis.

### 3.6. Determination of Cr^3+^ and Mn^2+^ Ions in Real Samples

The sensing ability of the developed LIBS-FP-AuNCs method was verified for real samples. We prepared tea broth and river water for Cr^3+^ and Mn^2+^ detection, respectively. The tea broth samples were prepared by adding 200 mL of boiling deionized water to 4 g tea leaves, soaking for 5 min, and filtering the residue. The river water samples were obtained from a canal in Beijing, China. The original concentrations of Cr and Mn in the tea broth and river water samples were obtained using ICP-MS. The Cr concentration in the tea broth sample was 5.20 μg L^−1^, and the Mn concentration in the river water sample was 0.147 μg L^−1^, both of which were below the minimum detectable concentrations. Thus, the samples were spiked with 0.02, 0.06, and 0.3 mg L^−1^ of heavy metal ions by adding a standard solution to the original samples. The analysis results are shown in Table 1. The recoveries of Cr^3+^ from the spiked tea broth ranged from 94.8% to 104.5%; the recoveries of Mn^2+^ from the spiked river water ranged from 98.8% to 105.1%. These results demonstrate that LIBS-FP-AuNCs were able to detect Cr^3+^ and Mn^2+^ in real samples and can be applied to the analysis of water samples with minimal interference from other ions. Moreover, this method can be applied to the analysis of tea broth samples without being affected by the sample color.

We also compared our method with the results of previous studies (Table 2). Our method is low-cost, rapid, simple, and highly sensitive, and it exhibited low detection limits and wide linear ranges for Cr^3+^ and Mn^2+^. The existing detection methods are mainly based on signal changes in the physical and chemical properties of nanoparticles caused by the heavy metals. However, these detection methods may be easily affected by factors such as the pH and the presence of other ions, leading to errors in the detection results. In addition, most of the nanoparticles used in traditional methods are in a liquid phase, and it is difficult to effectively separate them from the sample matrix after heavy metal adsorption. In contrast, our method successfully separated heavy metals by liquid–solid conversion by the FP-AuNCs, and then directly detected these using LIBS. Therefore, our method can reduce the impact of external interference factors on the detection results and achieve the direct removal of heavy metals, providing a more reliable and accurate method for detection.

## 4. Conclusions

In this work, we developed an effective LIBS-FP-AuNCs method for the rapid, simple, and sensitive detection of Cr^3+^ and Mn^2+^. The LODs for Cr^3+^ and Mn^2+^ were 7.5 and 9.0 μg L^−1^, respectively, which are lower than the Chinese standards for drinking water quality. Our method was also applied to real samples, for which the recoveries ranged from 94.6% to 105.1%. Moreover, this method offers unique advantages over current methods using nanomaterials. Firstly, AuNCs-modified filter paper has the ability to adsorb and enrich Cr^3+^ and Mn^2+^ from water samples, avoiding interference from other ions. Secondly, LIBS allows for the simultaneous detection of multiple heavy metals, thereby improving the detection efficiency. Notably, the color of the solution does not influence the accuracy of the method, making it applicable for various samples, such as tea, coffee, and soil extracts. Furthermore, with the rapid development of miniaturized LIBS equipment, the method is expected to achieve and guide rapid on-site detection of other heavy metals. In summary, this method has broad application prospects in industry, agriculture, and other fields.

## Figures and Tables

**Table 1:** Analysis of Cr and Mn in single-element spiked samples.

Real Sample	Sample No.	Target Ions (Original Concentration)	Added (mg L^−1^)	Founded (mg L^−1^)	Recovery
Tea broth	1	Cr (5.20 μg L^−1^)	0	-	-
	2		0.02	0.026 ±0.005	102.1%
	3		0.06	0.065 ±0.017	99.7%
	4		0.3	0.288 ±0.018	94.6%
River water	5	Mn (0.147 μg L^−1^)	0	-	-
	6		0.02	0.020 ±0.005	98.8%
	7		0.06	0.063 ±0.007	104.6%
	8		0.3	0.316 ±0.025	105.1%

**Table 2:** Comparison of our results with previous work.

Material	Existing Form	Target Ions	Detection Method	LOD	Ref.
GA-AuNPs	Liquid	Cr^3+^	Colorimetric	1.5 μM	[36]
DDS-AuNPs	Liquid	Cr^3+^	UV–vis spectra	0.78 μM	[37]
Rh6G-AuNPs	Liquid	Cr^3+^	Fluorescence	9.28 µM	[38]
AuNPs@3-mpa	Liquid	Cr^3+^	Electrochemistry	5.36 µM	[39]
AgNPs	Liquid	Mn^2+^	Colorimetric	0.5 µM	[40]
SiNPs	Liquid	Mn^2+^	UV–vis spectra	0.54 μM	[41]
Ti_3_C_2_ MXene nanosheets	Liquid	Mn^2+^	Fluorescence	0.11 μM	[42]
(Ag-Au)_mix_NPs	Liquid	Mn^2+^	Electrochemistry	8.42 μM	[43]
AuNCs	Solid	Cr^3+^	LIBS	0.15 μM	This work
		Mn^2+^		0.17 μM	

## Data Availability

The raw data supporting the conclusions of this article will be made available by the authors on request.
